# 4-(Diethyl­amino)­salicyl­aldehyde azine

**DOI:** 10.1107/S1600536811013237

**Published:** 2011-04-13

**Authors:** Jing-Bo Qiu, Bing-Zhu Yin

**Affiliations:** a Key Laboratory of Natural Resources of Changbai Mountain & Functional Molecules (Yanbian University), Ministry of Eduction, Yanji 133002, People’s Republic of China

## Abstract

The title compound, C_22_H_30_N_4_O_2_, has a crystallographic inversion center located at the mid-point of the N—N single bond. Apart from the four ethyl C atoms, the non-H atoms are nearly coplanar with a mean deviation of 0.0596 (2) Å. An intra­molecular O—H⋯N hydrogen bond occurs. In the crystal, weak inter­molecular C—H⋯O hydrogen bonds link the mol­ecules into layers parallel to (100).

## Related literature

For the synthesis, see Tang *et al.* (2009 ▶). For a related structure, see Gil *et al.* (2010 ▶). For applications of photochromic aromatic Schiff base mol­ecules as mol­ecular memories and switches, see Sliwa *et al.* (2005 ▶).
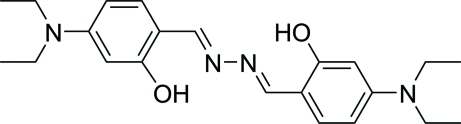


## Experimental

### 

#### Crystal data

C_22_H_30_N_4_O_2_
*M* *_r_* = 382.50
Monoclinic, 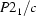

*a* = 8.736 (5) Å
*b* = 7.809 (5) Å
*c* = 16.122 (10) Å
β = 103.57 (2)°
*V* = 1069.1 (11) Å^3^
*Z* = 2
Mo *K*α radiation
μ = 0.08 mm^−1^
*T* = 290 K
0.15 × 0.14 × 0.12 mm

#### Data collection

Rigaku R-AXIS RAPID diffractometer
Absorption correction: multi-scan (*ABSCOR*; Higashi, 1995 ▶) *T*_min_ = 0.988, *T*_max_ = 0.991
9903 measured reflections
2431 independent reflections
1227 reflections with *I* > 2σ(*I*)
*R*_int_ = 0.046

#### Refinement

*R*[*F* ^2^ > 2σ(*F* ^2^)] = 0.074
*wR*(*F* ^2^) = 0.228
*S* = 1.10
2431 reflections
129 parameters
1 restraint
H-atom parameters constrained
Δρ_max_ = 0.45 e Å^−3^
Δρ_min_ = −0.25 e Å^−3^

### 

Data collection: *RAPID-AUTO* (Rigaku Corporation, 1998 ▶); cell refinement: *RAPID-AUTO*; data reduction: *CrystalStructure* (Rigaku/MSC and Rigaku Corporation, 2002 ▶); program(s) used to solve structure: *SHELXS97* (Sheldrick, 2008 ▶); program(s) used to refine structure: *SHELXL97* (Sheldrick, 2008 ▶); molecular graphics: *PLATON* (Spek, 2009 ▶); software used to prepare material for publication: *SHELXL97*.

## Supplementary Material

Crystal structure: contains datablocks global, I. DOI: 10.1107/S1600536811013237/ng5147sup1.cif

Structure factors: contains datablocks I. DOI: 10.1107/S1600536811013237/ng5147Isup2.hkl

Additional supplementary materials:  crystallographic information; 3D view; checkCIF report

## Figures and Tables

**Table 1:** Hydrogen-bond geometry (Å, °)

*D*—H⋯*A*	*D*—H	H⋯*A*	*D*⋯*A*	*D*—H⋯*A*
C8—H8*B*⋯O1^i^	0.97	2.64	3.481 (5)	145
O1—H1⋯N1	0.85	1.88	2.640 (3)	149

Symmetry code: (i) 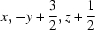.

**Fig. 1.**
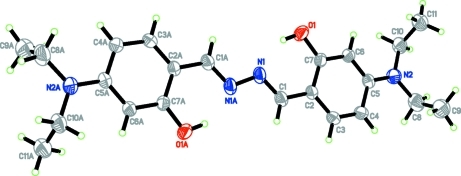
The crystal structure of the title compound, with the atom numbering. Displacement ellipsoids of non-H atoms are drawn at the 30% probalility level. [Symmetry code: A: 1 - x, 1 - y, 1 - z]

## supplementary crystallographic information

### Comment

Salicylaldehyde azine belongs to the photochromic aromatic schiff base molecules with two intramolecular hydrogen bonds (Gil *et al.*, 2010). The photochromism of the molecules, owing to enol-keto intramolecular tautomerism, attracts much interest because of possible applications, for example, in molecular memories and switches (Sliwa *et al.*, 2005). Herein, we report the crystal structure of the title compound.

The title compound, as shown in Fig. 1, all bond lengths and angles are in the normal ranges. Except for four carbon atoms, all the other non-hydrogen atoms nearly lie on the same plane. The intramolecular O—H···N and intermolecular C—H···O hydrogen bonds (Table 1) link the molecules into layers prallel to (100).

### Experimental

The title compound was prepared according to the literature (Tang *et al.*, 2009). Single crystals suitable for X-ray diffraction were prepared by slow evaporation a mixture of dichloromethane and petroleum (60–90 °C) at room temperature.

### Refinement

Carbon-bound H-atoms were placed in calculated positions (C—H 0.93 to 0.97 Å) and were included in the refinement in the riding model with *U*_iso_(H) = 1.2 or 1.5 *U*_eq_(C). The hydroxy H atom was located in a difference Fourier map and treated as riding on its parent O atom with *U*_iso_(H) = 1.5 *U*_eq_(O). The distance of O1 and H1 was restricted to 0.85 Å with *DFIX* command.

### Crystal data

**Table:**

C_22_H_30_N_4_O_2_	*F*(000) = 412
*M**_r_* = 382.50	*D*_x_ = 1.188 Mg m^−^^3^
Monoclinic, *P*2_1_/*c*	Mo *K*α radiation, λ = 0.71073 Å
Hall symbol: -P 2ybc	Cell parameters from 5162 reflections
*a* = 8.736 (5) Å	θ = 3.1–27.7°
*b* = 7.809 (5) Å	µ = 0.08 mm^−^^1^
*c* = 16.122 (10) Å	*T* = 290 K
β = 103.57 (2)°	Block, yellow
*V* = 1069.1 (11) Å^3^	0.15 × 0.14 × 0.12 mm
*Z* = 2	

### Data collection

**Table:**

Rigaku R-AXIS RAPID diffractometer	2431 independent reflections
Radiation source: fine-focus sealed tube	1227 reflections with *I* > 2σ(*I*)
graphite	*R*_int_ = 0.046
ω scans	θ_max_ = 27.5°, θ_min_ = 3.1°
Absorption correction: multi-scan (*ABSCOR*; Higashi, 1995)	*h* = −11→11
*T*_min_ = 0.988, *T*_max_ = 0.991	*k* = −10→10
9903 measured reflections	*l* = −20→20

### Refinement

**Table:**

Refinement on *F*^2^	Primary atom site location: structure-invariant direct methods
Least-squares matrix: full	Secondary atom site location: difference Fourier map
*R*[*F*^2^ > 2σ(*F*^2^)] = 0.074	Hydrogen site location: inferred from neighbouring sites
*wR*(*F*^2^) = 0.228	H-atom parameters constrained
*S* = 1.10	*w* = 1/[σ^2^(*F*_o_^2^) + (0.0919*P*)^2^ + 0.3133*P*] where *P* = (*F*_o_^2^ + 2*F*_c_^2^)/3
2431 reflections	(Δ/σ)_max_ = 0.003
129 parameters	Δρ_max_ = 0.45 e Å^−^^3^
1 restraint	Δρ_min_ = −0.25 e Å^−^^3^

### Special details

**Table:**

Experimental. (See detailed section in the paper)
Geometry. All e.s.d.'s (except the e.s.d. in the dihedral angle between two l.s. planes) are estimated using the full covariance matrix. The cell e.s.d.'s are taken into account individually in the estimation of e.s.d.'s in distances, angles and torsion angles; correlations between e.s.d.'s in cell parameters are only used when they are defined by crystal symmetry. An approximate (isotropic) treatment of cell e.s.d.'s is used for estimating e.s.d.'s involving l.s. planes.
Refinement. Refinement of *F*^2^ against ALL reflections. The weighted *R*-factor *wR* and goodness of fit *S* are based on *F*^2^, conventional *R*-factors *R* are based on *F*, with *F* set to zero for negative *F*^2^. The threshold expression of *F*^2^ > σ(*F*^2^) is used only for calculating *R*-factors(gt) *etc*. and is not relevant to the choice of reflections for refinement. *R*-factors based on *F*^2^ are statistically about twice as large as those based on *F*, and *R*- factors based on ALL data will be even larger.

### Fractional atomic coordinates and isotropic or equivalent isotropic displacement parameters (Å^2^)

**Table:**

	*x*	*y*	*z*	*U*_iso_*/*U*_eq_	
O1	0.2021 (2)	0.6394 (3)	0.57469 (13)	0.0809 (8)	
H1	0.2663	0.6244	0.5433	0.121*	
C1	0.4983 (3)	0.4599 (4)	0.60317 (18)	0.0556 (7)	
H1A	0.5914	0.3972	0.6165	0.067*	
C2	0.4129 (3)	0.4858 (3)	0.66828 (16)	0.0489 (7)	
C3	0.4698 (3)	0.4235 (4)	0.75059 (18)	0.0602 (8)	
H3	0.5638	0.3624	0.7625	0.072*	
C4	0.3937 (3)	0.4480 (4)	0.81492 (18)	0.0653 (9)	
H4	0.4360	0.4031	0.8688	0.078*	
C5	0.2503 (3)	0.5418 (4)	0.79966 (18)	0.0575 (7)	
C6	0.1898 (3)	0.5998 (4)	0.71712 (17)	0.0558 (7)	
H6	0.0939	0.6573	0.7048	0.067*	
C7	0.2678 (3)	0.5745 (4)	0.65257 (17)	0.0537 (7)	
C8	0.2343 (5)	0.5030 (6)	0.9510 (2)	0.0878 (11)	
H8A	0.3481	0.5128	0.9679	0.105*	
H8B	0.1906	0.5672	0.9915	0.105*	
C9	0.1893 (5)	0.3226 (6)	0.9530 (3)	0.1028 (14)	
H9A	0.0770	0.3142	0.9441	0.154*	
H9B	0.2387	0.2742	1.0074	0.154*	
H9C	0.2226	0.2614	0.9086	0.154*	
C10	0.0357 (4)	0.6857 (5)	0.8511 (2)	0.0735 (9)	
H10A	0.0461	0.7776	0.8123	0.088*	
H10B	0.0312	0.7373	0.9052	0.088*	
C11	−0.1157 (4)	0.5939 (5)	0.8161 (2)	0.0858 (11)	
H11A	−0.1144	0.5460	0.7615	0.129*	
H11B	−0.2018	0.6729	0.8100	0.129*	
H11C	−0.1283	0.5038	0.8545	0.129*	
N1	0.4511 (3)	0.5198 (3)	0.52728 (15)	0.0589 (7)	
N2	0.1766 (3)	0.5765 (4)	0.86429 (15)	0.0752 (8)	

### Atomic displacement parameters (Å^2^)

**Table:**

	*U*^11^	*U*^22^	*U*^33^	*U*^12^	*U*^13^	*U*^23^
O1	0.0839 (14)	0.1066 (19)	0.0616 (13)	0.0420 (13)	0.0362 (11)	0.0333 (12)
C1	0.0554 (15)	0.0532 (17)	0.0651 (18)	0.0019 (13)	0.0280 (13)	0.0001 (13)
C2	0.0482 (14)	0.0504 (16)	0.0521 (15)	−0.0016 (12)	0.0197 (11)	0.0012 (12)
C3	0.0454 (14)	0.076 (2)	0.0619 (18)	0.0117 (13)	0.0182 (13)	0.0085 (15)
C4	0.0519 (15)	0.097 (2)	0.0484 (16)	0.0105 (16)	0.0142 (12)	0.0101 (15)
C5	0.0505 (14)	0.0707 (19)	0.0569 (17)	0.0039 (13)	0.0242 (13)	0.0042 (14)
C6	0.0525 (14)	0.0620 (18)	0.0581 (16)	0.0110 (13)	0.0235 (13)	0.0091 (14)
C7	0.0560 (15)	0.0550 (17)	0.0549 (16)	0.0075 (13)	0.0227 (13)	0.0111 (13)
C8	0.084 (2)	0.115 (3)	0.074 (2)	0.002 (2)	0.0365 (19)	−0.009 (2)
C9	0.101 (3)	0.114 (4)	0.103 (3)	0.019 (3)	0.043 (2)	0.009 (2)
C10	0.075 (2)	0.083 (2)	0.072 (2)	0.0104 (18)	0.0374 (17)	−0.0018 (17)
C11	0.079 (2)	0.091 (3)	0.094 (3)	0.009 (2)	0.0336 (19)	0.007 (2)
N1	0.0643 (14)	0.0606 (15)	0.0616 (15)	0.0027 (12)	0.0345 (11)	0.0032 (12)
N2	0.0708 (16)	0.108 (2)	0.0548 (15)	0.0222 (15)	0.0301 (12)	0.0097 (14)

### Geometric parameters (Å, °)

**Table:**

O1—C7	1.351 (3)	C8—C9	1.465 (6)
O1—H1	0.8461	C8—N2	1.486 (4)
C1—N1	1.284 (4)	C8—H8A	0.9700
C1—C2	1.438 (4)	C8—H8B	0.9700
C1—H1A	0.9300	C9—H9A	0.9600
C2—C3	1.391 (4)	C9—H9B	0.9600
C2—C7	1.414 (4)	C9—H9C	0.9600
C3—C4	1.371 (4)	C10—N2	1.471 (4)
C3—H3	0.9300	C10—C11	1.494 (5)
C4—C5	1.422 (4)	C10—H10A	0.9700
C4—H4	0.9300	C10—H10B	0.9700
C5—N2	1.374 (3)	C11—H11A	0.9600
C5—C6	1.387 (4)	C11—H11B	0.9600
C6—C7	1.386 (3)	C11—H11C	0.9600
C6—H6	0.9300	N1—N1^i^	1.397 (4)
			
C7—O1—H1	107.9	C9—C8—H8B	109.4
N1—C1—C2	122.6 (3)	N2—C8—H8B	109.4
N1—C1—H1A	118.7	H8A—C8—H8B	108.0
C2—C1—H1A	118.7	C8—C9—H9A	109.5
C3—C2—C7	116.6 (2)	C8—C9—H9B	109.5
C3—C2—C1	121.1 (2)	H9A—C9—H9B	109.5
C7—C2—C1	122.3 (2)	C8—C9—H9C	109.5
C4—C3—C2	123.0 (3)	H9A—C9—H9C	109.5
C4—C3—H3	118.5	H9B—C9—H9C	109.5
C2—C3—H3	118.5	N2—C10—C11	114.4 (3)
C3—C4—C5	120.3 (3)	N2—C10—H10A	108.7
C3—C4—H4	119.8	C11—C10—H10A	108.7
C5—C4—H4	119.8	N2—C10—H10B	108.7
N2—C5—C6	121.5 (2)	C11—C10—H10B	108.7
N2—C5—C4	121.4 (3)	H10A—C10—H10B	107.6
C6—C5—C4	117.1 (2)	C10—C11—H11A	109.5
C7—C6—C5	122.0 (2)	C10—C11—H11B	109.5
C7—C6—H6	119.0	H11A—C11—H11B	109.5
C5—C6—H6	119.0	C10—C11—H11C	109.5
O1—C7—C6	117.9 (2)	H11A—C11—H11C	109.5
O1—C7—C2	121.2 (2)	H11B—C11—H11C	109.5
C6—C7—C2	120.9 (2)	C1—N1—N1^i^	114.3 (3)
C9—C8—N2	111.0 (3)	C5—N2—C10	122.0 (2)
C9—C8—H8A	109.4	C5—N2—C8	121.4 (3)
N2—C8—H8A	109.4	C10—N2—C8	116.6 (2)

Symmetry codes: (i) −*x*+1, −*y*+1, −*z*+1.

### Hydrogen-bond geometry (Å, °)

**Table:**

*D*—H···*A*	*D*—H	H···*A*	*D*···*A*	*D*—H···*A*
C8—H8B···O1^ii^	0.97	2.64	3.481 (5)	145
O1—H1···N1	0.85	1.88	2.640 (3)	149

Symmetry codes: (ii) *x*, −*y*+3/2, *z*+1/2.
